# Setting digital psychiatry in motion: towards dynamic digital markers for digital phenotyping

**DOI:** 10.1038/s44277-026-00059-y

**Published:** 2026-03-12

**Authors:** Axel Constant, C. Emre Koksal, Lena Palaniyappan

**Affiliations:** 1 School of Engineering and Informatics, The University of Sussex, Brighton, UK; 2 Department of Electrical and Computer Engineering, Ohio State University, Columbus, OH USA; 3 Douglas Mental Health University Institute, McGill University, Montreal, QC Canada

**Keywords:** Biomarkers, Computational models

## Abstract

Digital phenotyping uses data from smartphones and wearables to extract behavioural and biosocial markers of psychopathology in situ. Traditional entropy-based measures capture static system properties that neglect temporal dependencies critical to psychiatric phenomena. We propose a “dynamic” approach to the modelling of digital data capturing the time-varying aspects of processes of mental disorders. We defend that the resulting dynamic digital markers better capture variability in regulatory mechanisms of psychopathology.

## Lay Summary

Digital phenotyping uses information from smartphones and wearable devices to track patterns in behavior and physiology related to mental health. Most current methods summarize this data in ways that miss how experiences change over time. We propose a new, dynamic approach that focuses on how patterns evolve moment by moment. By capturing these changes, our method aims to better reflect how mental disorders involve shifting and unstable processes of regulation in everyday life.

## Introduction

Digital Phenotyping (DP) quantifies individual-level human markers of psychopathology using data from digital devices [1–3, 4] derived from data generated by processes configured across the scales of the biopsychosocial hierarchy. DP is interesting for psychiatry because it presents an opportunity to base clinical acts on markers that do not presuppose a bioreductive understanding of mental disorders [5] and opens the door for continuous, non-invasive sampling of behaviour, which has parallels in many medical fields (e.g., Holter monitors for heart rhythm, ambulatory glucose monitoring etc.).

Common DP methods use information theory [6]. Common measures include approximate entropy, sample entropy [7], permutation entropy [8], and multiscale entropy [9]. These are used to capture “static” properties: the *tendency* of a system to evolve or change. They do not directly measure time-varying “dynamic” properties: the temporal dependencies that unfold over time. Static measures provide a “snapshot” of a system’s complexity, regularity or predictability. They are valuable but insufficient to study psychiatric phenomena. DP’s focus on static measures is unsurprising, psychiatry traditionally leaning on static constructs (diagnoses, trait markers) failing to capture disorder progression.

This perspective argues for a “motion” approach that captures moment-to-moment flexibility, variability and predictability as a dynamic feature. The upshot of this approach is a framework for the production of “dynamic” digital markers (DDMs) that palliate the loss of dynamic information incurred by static measurements.

## Static vs. dynamic measurements

Healthcare measurements follow a hierarchical structure, moving from describing observable phenomena to understanding their underlying generative processes. At the first level, measurements at specific times (e.g., weight, heart rate) indicate a subject’s *state* (e.g., nutritional, cardiac health). Repeated and averaged measurements can reveal enduring states, often called *traits* in psychiatry. Both states and traits are descriptive ‘first-order’ measures that characterize surface-level, observable phenomena.

Then, regulatory mechanisms can be inferred from time-series data of these first order measures. For instance, information theoretic measures (e.g., heart rate entropy) are second order measures that capture moment-to-moment heartbeat fluctuations, providing a snapshot of autonomic heart rate regulation [10]. For physical illnesses, indexing intrinsic regulations via second-order measures predicts outcomes better [11–13], enabling patient selection and cardiac fitness and rehabilitation program titration [14]. But this is often insufficient for psychiatric illnesses. Time and context are crucial [15, 16], since it is the response of intrinsic regulatory processes to agentic action or social/environmental context (e.g., changes in ‘second order’ variability *per se*) that is of clinical interest in psychiatry.

Unlike medical diagnoses based on threshold-met criteria, psychiatric diagnoses explicitly incorporate time into their definition to differentiate phenomena of clinical interest from transient states (‘duration criteria’: 2-weeks for depression [17], 4-days for hypomania [18], etc.). A healthy individual’s mood variability might dip after a loss but then recover its normal ebb and flow within days, differentiating ‘acute’ vs. ‘remitted’ clinical macro-states. In depression, the recovery of this variability is prolonged or absent [19]: the system gets ‘stuck’ in the low complexity phase [20]. Other examples include situationally inappropriate autonomic responses in panic disorder [21], lack of behavioural flexibility in response to a changing social environment in personality disorders [22], bursts of cue-evoked impulsivity in eating disorders or addictions [23], to name a few.

Tracking how second-order variability changes over time and context can be done using *third-order* variables. Third-order variables are key to understanding how extrinsic factors interact and regulate latent intrinsic processes, this interaction being the substrate for interventions in psychiatry. The temporal dimension of variation in intrinsic processes across various contexts is of primary interest here, not a fixed state in health (i.e., first-order) or a snapshot of intrinsic variability (i.e., second-order) *per se* (see Table 1). Such third-order variables are the basis of DDMs.

**Table 1:** Orders of measures derived from time series data.

	Order	What it Measures	Examples	Limitation
**Static**	1^st^	States/traits: Value at a point in time or average value of a state/trait	Average time spent at home per week	Fails to capture variability
	2^nd^	States/traits variation: Capacity to exhibit variation in traits/states measured as complexity/regularity	Entropy of homestay over a month	Fails to capture response to context
**Dynamic**	3^rd^	Change in states/traits variation: Change over time of the capacity to exhibit variation in response to context	Entropy of the time taken for homestay to recover after a loss event	Computationally intensive

## DDM methodological guidelines

A DDM does not just ask the second order question of “What is the complexity of a patient’s behavior?” but address the third order one, of “How is this complexity generated and regulated?” We present a two steps methodological guideline in sufficiently general terms to allow researchers to implement DDMs using the models and formalism they see fit.

### The first step is information dynamics modelling

It involves describing a process as emitting a symbol stream and the information theoretic modelling of that process’ symbol stream, or message. One considers a stream of various symbols with (event) boundaries defined by external context conveyed by preceding symbols. Any method can be used to model the messages stream (e.g., Moving Approximate Entropy [24], Lempel-Ziv Complexity of sequences [25], block entropy [26]), so long as it allows extracting a context dependent temporal profile of information, or information dynamics.

One should then model the fluctuations in the message stream over time in a way that makes these fluctuations amenable to subsequent temporal dynamics modelling. Symbolic dynamic analysis^cf.^ [8] is one way to achieved this. It is used to analyse data from various phenomena of interest in biomedical contexts (e.g., cardiac signals [27]) and rests on the assessment of the ordered relation between data points rather than on the value of the data points themselves.

One can then explore the temporal properties of the underlying process of the data by modelling the temporal dynamics of the ordered rank using a computational modelling method, moving from a descriptive measure (first- or second-order) to an explicit, ‘third-order’ dynamic marker capturing the temporal dimension of variation in intrinsic processes across contexts, which is the primary substrate for psychiatric interventions.

### The second step is temporal dynamics modelling

It extracts temporal properties that are the basis of the DDMs. Here we suggest using a Markov chain (MC) as the model. Any model can be used, so long as one can derive from it the temporal properties of the data generating process. MCs model autonomous stochastic processes as transitions between states forming the state space of a system. They represent directly the temporal properties of the information dynamics for the data generating process, and indirectly, the underlying information source or process.

MCs derived DDMs can be tested as predictors of clinical variables or as mediators in a structural equation model. They indicate the time it takes for the digital data to through its characteristic patterns or increased how long it will take for the underlying process to reach a point where its probability of change approaches its steady state distribution [28]. MC derived DDMs provide quantitative, non-invasive proxies for the system’s adaptive capacity and flexibility, which is a cardinal feature of interest in mental disorders. For instance, a prolonged mixing time could serve as a proxy for the lack of behavioural flexibility in response to a changing social environment seen in personality disorders. Similarly, the recurrence time of a low-complexity state might quantify the inertia or inability to transition out of a maladaptive pattern, paralleling the ‘duration criteria’ explicitly incorporated into psychiatric diagnoses like depression or hypomania. Tracking these processes explicitly using digital phenotyping can help us understand contextual variations (or lack thereof) that define many mental disorders.

## A numerical proof of principle for DDMs

To help drive home the concept of DDM, we present a numerical proof of principle of temporal dynamics modelling illustrating the enhanced capacity of MC derived DDMs to discriminate between clinically relevant unobservable regulatory states responsible for variations in observed digital data. We provide additional details on our simulation in appendix.

**Fig. 1:**
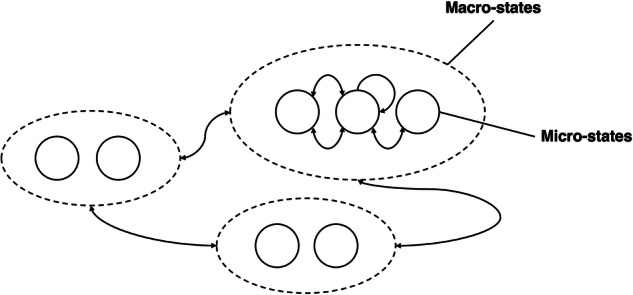
A sample multi-scale model for the patient states. Here, there are three macro-states with a varying number (2–3) of micro-states in each macro-state.

The system being modeled in this proof is a hypothetical patient’s day-by-day biobehavioral trajectory made of slow changing, latent macro-states (M) representing clinically interpretable macro-state of the patient’s clinical trajectory (e.g., acute symptom phase) made of fast changing micro-states (m) representing digitally observable biobehavioral traits (e.g., disrupted sleep patterns) expressed as an information dynamics. Narratively, this could represent a clinical participant in a digital phenotyping study periodically assessed by a psychiatrist, while continuously generating fast time scale digital data informing on the participants’ slower time scale clinical progression (Fig. 1).

**Fig. 2:**
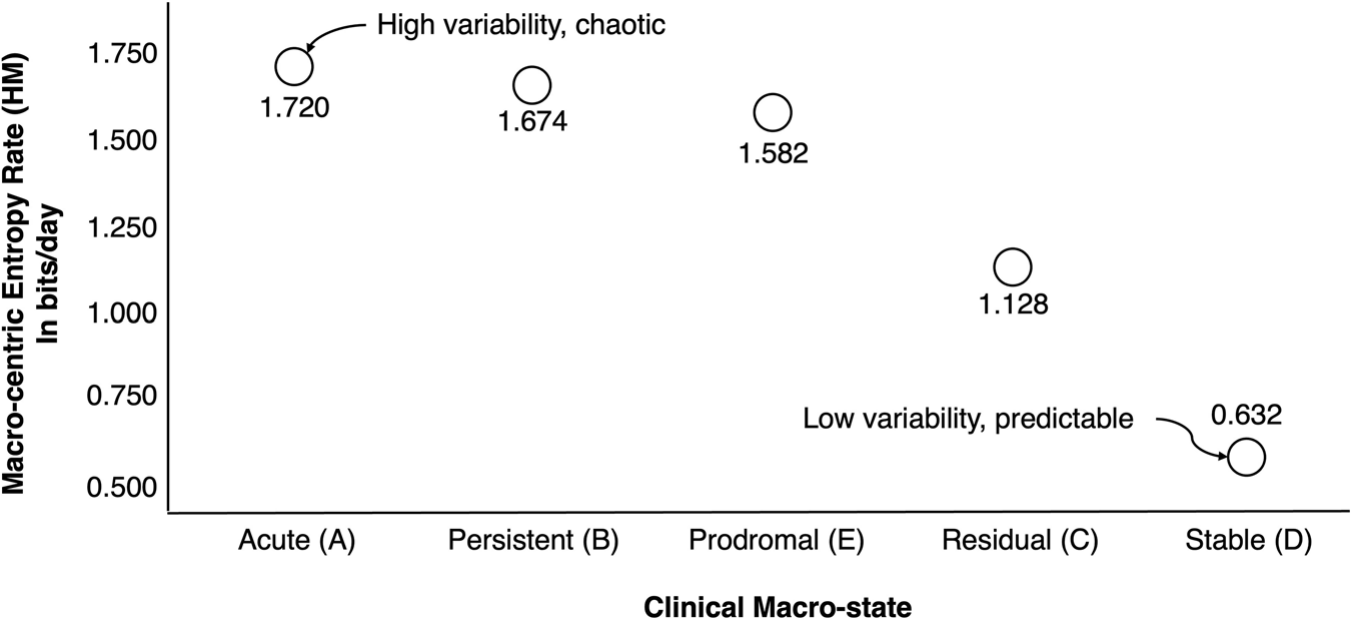
Macro-centric entropy rate per clinical macro-state. Visualization of results presented in Table 3.

We model the participant’s trajectory as a two-time-scale stochastic Markov Chain (MC) transitioning over macro and micro-states discretized into units of days. Macro states evolve on a scale of weeks to months thereby reflecting illness progression, whereas microstates evolve daily. The transition probabilities of microstates depend on the macro-state the system is at, such that multiple microstates can be visited when in a same macro state (e.g., multiple actigraphy reports within the time spent of a single acute episode) (see Fig.2).

We model 5 macro-states – acute, persistent, residual, stable, prodromal {A,B,C,D,E} -- and 4 digital sensor microstates regulated routine, withdrawn/no activity, no regular usage (indicating disturbance), fragmented activity (frequent shifts) {R,W,N,F}. The resulting flattened Markov chain consists of 5 × 4 = 20 states, each identified by a macro–micro pair (M,m). Consequently, we have a 20 × 20 matrix mapping the transition between 20 pairs of states (M,m) from day_t-1 to day_t.

We parameterized the transition matrix to reflect a clinically realistic trajectory. For an illness such as psychosis, after a substantial “prodromal” period, preceding in a “acute” macro-state (e.g., needing hospitalisation), the system is more likely to spend time in a “stable” (no active symptoms) or “residual” macro states (low symptom phase) than in a “persistent” (non-responsive phase), all the while frequently transitioning between microstates (fluctuating daily symptoms/signs) within the same macro state. This yields the transition probability matrix in Table 2.

**Table 2:** Asymmetric two-time-scale transition matrix P (one transition per day). States are ordered by macro-state blocks A–E, each containing micro-states R,W,N,F. Vertical rules indicate block structure (macro changes are rare).

	AR	AW	AN	AF	BR	BW	BN	BF	CR	CW	CN	CF	DR	DW	DN	DF	ER	EW	EN	EF
**AR**	0.2087	0.1896	0.1133	0.0496	0.0005	0.0001	0.0001	0.0000	0.0011	0.0005	0.0002	0.0002	0.0002	0.0001	0.0000	0.0000	0.0359	0.0216	0.0108	0.0036
**AW**	0.0092	0.3280	0.1181	0.0248	0.0005	0.0001	0.0001	0.0000	0.0011	0.0005	0.0002	0.0002	0.0002	0.0001	0.0000	0.0000	0.0359	0.0216	0.0108	0.0036
**AN**	0.0669	0.1642	0.2764	0.0521	0.0005	0.0001	0.0001	0.0000	0.0011	0.0005	0.0002	0.0002	0.0002	0.0001	0.0000	0.0000	0.0359	0.0216	0.0108	0.0036
**AF**	0.0779	0.0577	0.1383	0.2860	0.0005	0.0001	0.0001	0.0000	0.0011	0.0005	0.0002	0.0002	0.0002	0.0001	0.0000	0.0000	0.0359	0.0216	0.0108	0.0036
**BR**	0.0005	0.0005	0.0003	0.0001	0.2348	0.1596	0.0758	0.0267	0.0013	0.0006	0.0003	0.0002	0.0001	0.0001	0.0000	0.0000	0.0414	0.0237	0.0118	0.0039
**BW**	0.0005	0.0005	0.0003	0.0001	0.0915	0.2786	0.0746	0.0199	0.0013	0.0006	0.0003	0.0002	0.0001	0.0001	0.0000	0.0000	0.0414	0.0237	0.0118	0.0039
**BN**	0.0005	0.0005	0.0003	0.0001	0.0558	0.1304	0.2963	0.0561	0.0013	0.0006	0.0003	0.0002	0.0001	0.0001	0.0000	0.0000	0.0414	0.0237	0.0118	0.0039
**BF**	0.0005	0.0005	0.0003	0.0001	0.0748	0.0505	0.1115	0.3024	0.0013	0.0006	0.0003	0.0002	0.0001	0.0001	0.0000	0.0000	0.0414	0.0237	0.0118	0.0039
**CR**	0.0001	0.0001	0.0001	0.0000	0.0003	0.0001	0.0001	0.0000	0.4834	0.1190	0.0552	0.0330	0.0275	0.0072	0.0036	0.0018	0.0331	0.0189	0.0094	0.0031
**CW**	0.0001	0.0001	0.0001	0.0000	0.0003	0.0001	0.0001	0.0000	0.2341	0.3129	0.0622	0.0214	0.0275	0.0072	0.0036	0.0018	0.0331	0.0189	0.0094	0.0031
**CN**	0.0001	0.0001	0.0001	0.0000	0.0003	0.0001	0.0001	0.0000	0.1272	0.1297	0.3580	0.0723	0.0275	0.0072	0.0036	0.0018	0.0331	0.0189	0.0094	0.0031
**CF**	0.0001	0.0001	0.0001	0.0000	0.0003	0.0001	0.0001	0.0000	0.1730	0.0731	0.1175	0.3236	0.0275	0.0072	0.0036	0.0018	0.0331	0.0189	0.0094	0.0031
**DR**	0.0000	0.0000	0.0000	0.0000	0.0001	0.0000	0.0000	0.0000	0.0007	0.0003	0.0001	0.0001	0.6591	0.1142	0.0422	0.0235	0.0840	0.0504	0.0252	0.0084
**DW**	0.0000	0.0000	0.0000	0.0000	0.0001	0.0000	0.0000	0.0000	0.0007	0.0003	0.0001	0.0001	0.3022	0.3787	0.0503	0.0220	0.0840	0.0504	0.0252	0.0084
**DN**	0.0000	0.0000	0.0000	0.0000	0.0001	0.0000	0.0000	0.0000	0.0007	0.0003	0.0001	0.0001	0.2052	0.1350	0.4357	0.0452	0.0840	0.0504	0.0252	0.0084
**DF**	0.0000	0.0000	0.0000	0.0000	0.0001	0.0000	0.0000	0.0000	0.0007	0.0003	0.0001	0.0001	0.2477	0.0639	0.1156	0.4514	0.0840	0.0504	0.0252	0.0084
**ER**	0.0014	0.0013	0.0008	0.0004	0.0012	0.0003	0.0002	0.0001	0.0011	0.0005	0.0002	0.0002	0.0250	0.0065	0.0032	0.0011	0.4126	0.2027	0.0894	0.0292
**EW**	0.0014	0.0013	0.0008	0.0004	0.0012	0.0003	0.0002	0.0001	0.0011	0.0005	0.0002	0.0002	0.0250	0.0065	0.0032	0.0011	0.2196	0.3492	0.0898	0.0634
**EN**	0.0014	0.0013	0.0008	0.0004	0.0012	0.0003	0.0002	0.0001	0.0011	0.0005	0.0002	0.0002	0.0250	0.0065	0.0032	0.0011	0.1098	0.2026	0.2812	0.0686
**EF**	0.0014	0.0013	0.0008	0.0004	0.0012	0.0003	0.0002	0.0001	0.0011	0.0005	0.0002	0.0002	0.0250	0.0065	0.0032	0.0011	0.1317	0.1040	0.1341	0.2898

A first order metric of the MC is its macro-states steady state distribution [(acute A) 0.0532; (persistent B) 0.0338; (residual C) 0.1249; (stable D) 0.5959; (prodromal E) 0.1923]. This gives us descriptive information, that the stable state D accounts for 60% of the probability of occupancy of macro-state, with much of the remainder of the probability being captured by the prodromal and residual macro-state.

A second order metric of the MC could be its entropy rate [29] or the entropy of the MC’s transitions distributions weighted by its steady state distribution, such that

1 $$H=-{\sum}_{i\in S}{\pi }_{i}{\sum}_{j\in S}{P}_{{ij}}{log }_{2}{P}_{{ij}}$$

which,

2 $${\pi }_{i}={{\lim}}_{{{\rm{t}}}\longrightarrow \infty }{\Pr ({X}_{t}=i)}$$

which is approximately 1.0 (bits/day). Entropy rate aggregates variability across all macro-states and micro-states, thereby averaging out information about the hidden heterogeneity in regulatory mechanisms underlying macro-states. While first and second order metrics summarize long-run occupancy and overall behavioral variability, they are insufficient for differentiating macro-states.

A third order metric is “macro-state–centric” entropy rates, which is obtained by calculating the entropy rate of the micro-state dynamics for a given macro state taken in isolation. This requires conditioning a given macro-state (e.g, A) of interest onto the remaining macro states to extract a conditional 4 × 4 transition matrix ${\widetilde{P}}^{(M)}$ with stationary distribution ${\widetilde{\pi }}^{(M)}$. Macro-state–centric entropy rates is defined as

3 $${H}_{M}\triangleq -{\sum}_{i\in {S}_{m}}{\widetilde{\pi }}_{i}^{\left(M\right)}{\sum}_{j\in {S}_{m}}{\widetilde{P}}_{{ij}}^{\left(M\right)}{log }_{2}{\widetilde{P}}_{{ij}}^{\left(M\right)}$$

Where ${S}_{m}$ is the set of microstates {R,W,N,F}. Hm is the entropy rate of micro sates dynamics assuming no change in macro sate. This effectively isolates short-term behavioral variability from slow macro-level transitions, thereby capturing how tightly or loosely behavior is regulated within a macro-sate regime. This yields 1.720 Hm for acute (A), 1.674 Hm for persistent (B) 1.128 hm for residual (C), 0.632 for stable (D), and 1.582 for prodromal (E).

Effectively, Hm captures how entropy rate – the second order variable – varies over time within a single macro-state. Macro-centric entropy rates provide a strong discriminator of latent clinical regime despite substantial overlap in steady-state probabilities and global entropy measures. DDMs based on such third-order variables would capture differences in the regulation of variability itself, rather than variability or occupancy alone.

Additionally, macro-centric entropy rates are realistic and feasible given typical data constraints in digital phenotyping. They can be approximated empirically from finite observation windows and with few data points. For a given time series, micro-state trajectories can be segmented into windows of length T days that are fully contained within the occupancy time of a given macro-state. Micro-state transition probabilities can then be estimated empirically within each window, and entropy rates can be computed from the resulting transition matrices using a standard plug-in procedure.

This involves (i) deriving an M,m X M,m empirical transition matrix from observed transition frequencies of M,m over a given time window a length T, (ii) computing the macro-centric entropy rate based on that transition matrix, and (iii) repeating the process by moving the time window further down the time series. As one moves the time window, the mean values and standard errors of the resulting empirical distribution of macro-centric entropy rate estimates approach their ground truth, or theoretical values (see Table 3 for T ∈{15,30,90}days and appendix for details).

**Table 3:** Empirical macro-centric entropy rates (bits/day) for finite window lengths T, compared with theoretical values.

Macro-state	Theoretical	*T* = 15 days	*T* = 30 days	*T* = 90 days
Acute (A)	1.720	1.123 ± 0.031	1.413 ± 0.015	1.638 ± 0.007
Persistent (B)	1.674	1.128 ± 0.027	1.362 ± 0.019	1.557 ± 0.011
Residual (C)	1.128	0.925 ± 0.032	0.954 ± 0.014	1.040 ± 0.007
Stable (D)	0.632	0.749 ± 0.037	0.691 ± 0.014	0.613 ± 0.005
Prodromal (E)	1.582	1.015 ± 0.018	1.251 ± 0.010	1.465 ± 0.006

Table 3 shows that even for relatively short windows (e.g., T = 15 days), there is a clear separation between the stable well (D) and acute symptomatic (A) macro-states. Figure 2 plots these results. For longer windows (e.g., T ≥ 30 days), empirical entropy-rate estimates stabilize sufficiently to distinguish all macro-states reliably. This confirms that third-order metrics for dynamic digital markers are not only theoretically well-founded, but also practically estimable from realistic observation horizons encountered in digital phenotyping studies.

In practical deployment, microstates may not be directly observed, or may only be observed infrequently – e.g., only during clinical assessment – such that one could not obtain a M,m X M,m transition matrix, and only an m X m one. But our proof of principle shows that sliding window entropy rates approximates macro-centric entropy rates, the continuous monitoring of which should therefore provide a means of detecting macro-state changes in real time. This means that even though one cannot observe patient specific macro-states prior to deployment, one can initialize the model with population level priors and refined them with idiosyncratic data over time (see appendix for details). Additional details on data collection and annotation pipelines to produce DDMs is beyond the scope of this conceptual paper.

This allows macro-state changes to be detected without explicit state labels, enabling adaptive, data-driven identification of clinically meaningful transitions as they unfold. For instance, sustained deviations from baseline entropy levels or consistent trends toward the characteristic entropy range of another macro-state can be interpreted as early indicators of a regime shift. In practice, standard change-point detection methods applied to the time series of sliding-window entropy-rate estimates can be used to automatically identify statistically significant shifts, providing a principled mechanism for detecting macro-state transitions in real time.

## Towards DDMs for DP

To fully realize DP’s potential in psychiatry, its markers must transition from marking state/trait (static) to marking the process (dynamic). Collapsing a patient’s daily complexity into a single summary value miss critical regulatory information. Methods along the lines of those gestured towards in this paper are candidate for addressing this limitation of DP. The next step is for the community to learn how to best apply this method to real clinical dataset, and moving beyond characterization by showing that DDMs predict relapse, treatment response, or functional outcomes better than static markers. For digital phenotyping to fulfill its promise, it must evolve from static to dynamic analysis, from snapshots to trajectories, from traits to processes. This will align psychiatry with the rest of medicine in continuous monitoring and better capture the living course of human suffering.

## Supplementary information

Appendix
