# Supplementary material for: Setting digital psychiatry in motion: towards dynamic digital markers for digital phenotyping
Source: NPP Digit Psychiatry Neurosci. 2026 Mar 12;4:6. doi: 10.1038/s44277-026-00059-y (PMC12982652; doi:10.1038/s44277-026-00059-y)
Supplement: Supplementary file 1 — Appendix [file 44277_2026_59_MOESM1_ESM.docx]

1. Appendix - Numerical Evaluation of Third-Order Digital Markers

# Objective

The objective of this section is to provide the details of our evaluation for the efficacy of *third-order digital markers* for identifying and characterizing latent psychiatric regimes. We contrast these markers with conventional first- and second-order digital metrics commonly used in digital phenotyping. Specifically, we assess whether *macro-state–centric entropy rates*, defined as entropy rates of short-term behavioral dynamics conditioned on a latent clinical macro-state, provide improved discriminability of clinically meaningful patient states. As mentioned in the previous section, our third-order metrics are de- signed to capture how behavioral variability itself is regulated over time, rather than static levels of behavior or aggregate variability alone.

# Numerical Setup and State-Space Model

We consider a two-time-scale stochastic model motivated by real-world psychiatric trajectories as observed through passive digital sensing. Time is discretized in units of days, with one state transition per day. A sample Markov chain is illustrated in Fig. [1.2,](#_bookmark0) in which the macro-states are dashed ellipses, within each there are a number of micro-states.

macro-states


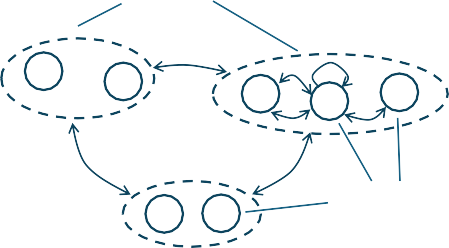


micro-states

Figure 1: A sample multi-scale model for the patient states. Here, there are three macro-states with a varying number (2-3) of micro-states in each macro-state.

In our model, at the slower time-scale, the patient occupies one of five latent *macro-states*, representing clinically interpretable regimes. These macro-states evolve over weeks to months and reflect sustained clinical conditions rather than daily fluctuations. Table [1](#_bookmark1) summarizes the macro- states used in the numerical evaluation.

At the faster time-scale, each macro-state contains four *micro-states* capturing short-term digital behavioral motifs observable through daily digital markers. These micro-states are shared across all macro-states, but their transition dynamics and occupancy probabilities depend on the current macro-state. Table [2](#_bookmark2) summarizes the micro-state definitions.

The resulting flattened Markov chain consists of 5 *×* 4 = 20 states, each identified by a macro– micro pair (*M, m*). Transition probabilities are chosen to reflect real-world clinical intuition: in- dividuals spend substantially more time in the stable well macro-state than in acute symptomatic states, while micro-state transitions occur frequently within a given macro-state.

The associated 20 *×* 20 transition matrix we have chosen is given in Table [3.](#_bookmark3) We chose the the transitions between micro-states at a given macro-state to be frequent, with a transition occurring at a time scale of days, while transitions from one macro-state to another occurs with much smaller

Table 1: Macro-state definitions and identifiers.

| ID | Macro-state | Clinical interpretation |
| --- | --- | --- |
| A | Acute symptomatic | Severe symptom burden with marked functional im- |
|  |  | pairment; high instability and dysregulation. |
| B | Persistent symptomatic | Chronic or residual symptomatology with limited vari- |
|  |  | ability and slow recovery dynamics. |
| C | Improving/early recovery | Active symptom improvement with increasing behav- |
|  |  | ioral regularity and flexibility. |
| D | Stable well | Euthymic baseline with structured routines, stable |
|  |  | functioning, and low relapse risk. |
| E | At-risk/prodromal | Subclinical deterioration or early warning phase pre- |
|  |  | ceding relapse, with emerging irregularities. |

Table 2: Micro-state definitions and identifiers (shared across macro-states).

| ID | Micro-state | Behavioral interpretation |
| --- | --- | --- |
| R | Regulated routine | Regular sleep–wake timing, moderate device use, and |
|  |  | structured daily activity. |
| W | Withdrawn/low engagement | Reduced communication and mobility, increased |
|  |  | home-stay, and low interaction diversity. |
| N | Circadian disruption | Late-night or irregular device use indicative of sleep |
|  |  | disturbance or rumination. |
| F | Fragmented/agitated use | Frequent short sessions, rapid app switching, and er- |
|  |  | ratic engagement patterns. |

probabilities. Hence, there is a much longer holding time (of order months, as we shall see) for each macro-state.

# Baseline First- and Second-Order Metrics

As baseline comparators, we report standard first-order and second-order digital metrics, including steady-state macro-state probabilities and global entropy rates of the full 20-state Markov chain.

Table [4](#_bookmark4) reports the steady-state probabilities of the five macro-states under the full 20-state Markov model. As intended by construction, the *Stable well* macro-state dominates the stationary distribution, accounting for approximately 60% of long-run occupancy, while *Acute* and *Persistent* states are comparatively rare. The *At-risk* and *Improving* macro-states together capture a substan- tial fraction of time, reflecting clinically plausible intermediate regimes. While these steady-state probabilities provide a useful population-level characterization, they are intrinsically retrospective and do not offer a reliable indicator of the patient’s current macro-state at any given time.

At the second order, we will calculate the **entropy rate** of the chain. For a discrete-time ergodic Markov chain on a finite state space *S* with transition matrix *P* and stationary distribution *π*, the entropy rate of the chain is identical to

$H=-\sum_{i\in\mathcal{S}} \pi_{i}\sum_{j\in\mathcal{S}} P_{\mathrm{ij}} \log_{2} P_{\mathrm{ij}},$ (1)

where *π_i_* = lim*_t→∞_* Pr(*X_t_* = *i*).

Table 3: Asymmetric two-time-scale transition matrix *P* (one transition per day). States are ordered by macro-state blocks A–E, each containing micro-states R,W,N,F. Vertical rules indicate block structure (macro changes are rare).

|  | AR | AW | AN | AF | BR | BW | BN | BF | CR | CW | CN | CF | DR | DW | DN | DF | ER | EW | EN | EF |
| --- | --- | --- | --- | --- | --- | --- | --- | --- | --- | --- | --- | --- | --- | --- | --- | --- | --- | --- | --- | --- |

| AR | 0.2087 0.1896 0.1133 0.0496 | 0.0005 0.0001 0.0001 0.0000 | 0.0011 0.0005 0.0002 0.0002 | 0.0002 0.0001 0.0000 0.0000 | 0.0359 0.0216 0.0108 0.0036 |
| --- | --- | --- | --- | --- | --- |
| AW | 0.0902 0.3280 0.1181 0.0248 | 0.0005 0.0001 0.0001 0.0000 | 0.0011 0.0005 0.0002 0.0002 | 0.0002 0.0001 0.0000 0.0000 | 0.0359 0.0216 0.0108 0.0036 |
| AN | 0.0669 0.1642 0.2764 0.0521 | 0.0005 0.0001 0.0001 0.0000 | 0.0011 0.0005 0.0002 0.0002 | 0.0002 0.0001 0.0000 0.0000 | 0.0359 0.0216 0.0108 0.0036 |
| AF | 0.0779 0.0577 0.1383 0.2860 | 0.0005 0.0001 0.0001 0.0000 | 0.0011 0.0005 0.0002 0.0002 | 0.0002 0.0001 0.0000 0.0000 | 0.0359 0.0216 0.0108 0.0036 |

| BR | 0.0005 0.0005 0.0003 0.0001 | 0.2348 0.1596 0.0758 0.0267 | 0.0013 0.0006 0.0003 0.0002 | 0.0001 0.0001 0.0000 0.0000 | 0.0414 0.0237 0.0118 0.0039 |
| --- | --- | --- | --- | --- | --- |
| BW | 0.0005 0.0005 0.0003 0.0001 | 0.0915 0.2786 0.0745 0.0199 | 0.0013 0.0006 0.0003 0.0002 | 0.0001 0.0001 0.0000 0.0000 | 0.0414 0.0237 0.0118 0.0039 |
| BN | 0.0005 0.0005 0.0003 0.0001 | 0.0558 0.1304 0.2963 0.0561 | 0.0013 0.0006 0.0003 0.0002 | 0.0001 0.0001 0.0000 0.0000 | 0.0414 0.0237 0.0118 0.0039 |
| BF | 0.0005 0.0005 0.0003 0.0001 | 0.0748 0.0505 0.1115 0.3024 | 0.0013 0.0006 0.0003 0.0002 | 0.0001 0.0001 0.0000 0.0000 | 0.0414 0.0237 0.0118 0.0039 |

| CR | 0.0001 0.0001 0.0001 0.0000 | 0.0003 0.0001 0.0001 0.0000 | 0.4834 0.1190 0.0552 0.0330 | 0.0275 0.0072 0.0036 0.0018 | 0.0331 0.0189 0.0094 0.0031 |
| --- | --- | --- | --- | --- | --- |
| CW | 0.0001 0.0001 0.0001 0.0000 | 0.0003 0.0001 0.0001 0.0000 | 0.2341 0.3129 0.0622 0.0214 | 0.0275 0.0072 0.0036 0.0018 | 0.0331 0.0189 0.0094 0.0031 |
| CN | 0.0001 0.0001 0.0001 0.0000 | 0.0003 0.0001 0.0001 0.0000 | 0.1272 0.1297 0.3580 0.0723 | 0.0275 0.0072 0.0036 0.0018 | 0.0331 0.0189 0.0094 0.0031 |
| CF | 0.0001 0.0001 0.0001 0.0000 | 0.0003 0.0001 0.0001 0.0000 | 0.1730 0.0731 0.1175 0.3236 | 0.0275 0.0072 0.0036 0.0018 | 0.0331 0.0189 0.0094 0.0031 |

| DR | 0.0000 0.0000 0.0000 0.0000 | 0.0001 0.0000 0.0000 0.0000 | 0.0007 0.0003 0.0001 0.0001 | 0.6591 0.1142 0.0422 0.0235 | 0.0840 0.0504 0.0252 0.0084 |
| --- | --- | --- | --- | --- | --- |
| DW | 0.0000 0.0000 0.0000 0.0000 | 0.0001 0.0000 0.0000 0.0000 | 0.0007 0.0003 0.0001 0.0001 | 0.3022 0.3787 0.0503 0.0220 | 0.0840 0.0504 0.0252 0.0084 |
| DN | 0.0000 0.0000 0.0000 0.0000 | 0.0001 0.0000 0.0000 0.0000 | 0.0007 0.0003 0.0001 0.0001 | 0.2052 0.1350 0.4357 0.0452 | 0.0840 0.0504 0.0252 0.0084 |
| DF | 0.0000 0.0000 0.0000 0.0000 | 0.0001 0.0000 0.0000 0.0000 | 0.0007 0.0003 0.0001 0.0001 | 0.2477 0.0639 0.1156 0.4514 | 0.0840 0.0504 0.0252 0.0084 |

| ER | 0.0014 0.0013 0.0008 0.0004 | 0.0012 0.0003 0.0002 0.0001 | 0.0011 0.0005 0.0002 0.0002 | 0.0250 0.0065 0.0032 0.0011 | 0.4126 0.2027 0.0894 0.0292 |
| --- | --- | --- | --- | --- | --- |
| EW | 0.0014 0.0013 0.0008 0.0004 | 0.0012 0.0003 0.0002 0.0001 | 0.0011 0.0005 0.0002 0.0002 | 0.0250 0.0065 0.0032 0.0011 | 0.2196 0.3492 0.0898 0.0234 |
| EN | 0.0014 0.0013 0.0008 0.0004 | 0.0012 0.0003 0.0002 0.0001 | 0.0011 0.0005 0.0002 0.0002 | 0.0250 0.0065 0.0032 0.0011 | 0.1098 0.2026 0.2812 0.0686 |
| EF | 0.0014 0.0013 0.0008 0.0004 | 0.0012 0.0003 0.0002 0.0001 | 0.0011 0.0005 0.0002 0.0002 | 0.0250 0.0065 0.0032 0.0011 | 0.1317 0.1040 0.1341 0.2898 |

Table 4: Steady-state macro-state probabilities.

| macro-state | Steady-state probability |
| --- | --- |
| Acute (A) | 0.0532 |
| Persistent (B) | 0.0338 |
| Improving (C) | 0.1249 |
| Stable well (D) | 0.5959 |
| At-risk (E) | 0.1923 |
| Sum | 1.0000 |

The Markov chain described by *P* given in Table [3,](#_bookmark3) the entropy rate can be calculated to be

*H ≈* 1*.*0 bits/day*,*

This global entropy rate aggregates variability across all macro-states and microstates, effectively averaging over clinically distinct regimes. As a result, it obscures the heterogeneity of behavioral regulation across macro-states: markedly different clinical conditions can yield similar contribu- tions to the global entropy rate. Consequently, neither steady-state occupancy nor global entropy provides sufficient resolution for macro-state identification or monitoring at the individual level.

While these metrics summarize long-run occupancy and overall behavioral variability, they are insufficient for identifying the patient’s current macro-state. In particular, distinct macro- states may exhibit similar steady-state probabilities or comparable global entropy values, thereby obscuring clinically meaningful differences in how behavioral variability is structured and regulated over time.

# Third-Order Results: Macro-Centric Entropy Rates

To obtain third-order digital markers, we compute *macro-centric entropy rates* by isolating the micro-state dynamics within each macro-state and evaluating the entropy rate of the corresponding conditional micro-scale Markov chain.

For a given macro-state *M ∈ {A, B, C, D, E}*, let *P* ^(^*^M^*^)^ denote the 4 *×* 4 conditional micro-state transition matrix obtained by conditioning on remaining in macro-state *M* , and let *π*˜^(^*^M^*^)^ be its stationary distribution. The macro-centric entropy rate (in bits/day) is defined as:

-

$H_{M}\triangleq-\sum_{i\in\mathcal{S}_{\mathcal{m}}} \tilde{\pi_{i}^{\left( M \right)}}\sum_{j\in\mathcal{S}_{\mathcal{m}}} \tilde{P_{\mathrm{ij}}^{\left( M \right)}} \log_{2} \tilde{P_{\mathrm{ij}}^{\left( M \right)}},$ (2)

where *S_m_* = *{R, W, N, F}* denotes the set of micro-states.

The quantity *H_M_* measures the average uncertainty in next-day micro-state behavior when the patient remains in macro-state *M* , averaged with respect to the long-run micro-state occupancy within that macro-state. By conditioning on macro-state persistence, *H_M_* isolates short-term be- havioral variability from slow macro-level transitions, thereby capturing how tightly or loosely behavior is regulated within a given clinical regime. Since each macro-state contains four micro- states, the entropy rate is bounded as 0 *≤ H_M_ ≤* log_2_ 4 = 2 bits/day.

Table [5](#_bookmark5) reports the entropy rates of the micro-state dynamics conditioned on each macro-state.

Table 5: Macro-centric entropy rates (third-order digital markers).

| macro-state | Entropy rate (bits/day) |
| --- | --- |
| Acute (A) | 1.720 |
| Persistent (B) | 1.674 |
| Improving (C) | 1.128 |
| Stable well (D) | 0.632 |
| At-risk (E) | 1.582 |

These results reveal a clear and clinically meaningful ordering of macro-states that is not appar- ent from first- or second-order metrics alone. The *Acute* macro-state exhibits the highest entropy rate, reflecting highly irregular and weakly regulated short-term behavior. The *Persistent* and *At- risk* macro-states also show elevated entropy, consistent with ongoing dysregulation and instability, albeit less extreme than in acute episodes. In contrast, the *Stable well* macro-state is characterized by a markedly lower entropy rate, indicative of structured, predictable daily routines and strong behavioral regulation. The *Improving* macro-state occupies an intermediate position, reflecting partial recovery of regularity and control.

Importantly, these macro-centric entropy rates provide a strong discriminator of latent clinical regime despite substantial overlap in steady-state probabilities and global entropy measures. This demonstrates that third-order digital markers capture differences in the *regulation of variability* it- self, rather than variability or occupancy alone, making them particularly well-suited for identifying and tracking clinically relevant patient states.

These third-order metrics exhibit clear and systematic separation across macro-states. In partic- ular, the stable well macro-state (D) is characterized by a markedly lower entropy rate, reflecting structured and predictable daily routines. In contrast, the acute symptomatic macro-state (A) exhibits substantially higher entropy rates, indicative of fragmented and dysregulated behavior. Intermediate macro-states (B, C, and E) occupy ordered positions between these extremes.

These results demonstrate that third-order entropy rates provide a natural and clinically inter- pretable discriminator of latent patient state, capturing differences in regulatory dynamics that are not accessible through first- or second-order metrics alone.

# Practical Estimation of Macro-state Entropy Rates from Finite Time-Windows

In practical settings, macro-centric entropy rates can be approximated empirically from finite ob- servation windows. Digital trajectories are segmented into windows of length *T* days that are fully contained within a given macro-state sojourn. Micro-state transition probabilities are estimated empirically within each window, and entropy rates are computed from the resulting transition matrices.

To estimate entropy rates from finite observations, we employ a standard plug-in procedure applied to short windows of the observed state sequence. For a given window of length *T* days, we first record the number of observed transitions between each pair of states within the window and normalize these counts to obtain an empirical transition matrix. The stationary distribution associated with this empirical transition matrix is then computed, and the entropy rate is evaluated as the average one-step uncertainty under this stationary distribution. For macro-centric estimates, this procedure is applied only to windows fully contained within a single macro-state sojourn, thereby isolating short-term micro-scale behavioral dynamics from slower macro-level transitions. Repeating this procedure across multiple windows yields an empirical distribution of entropy-rate estimates, from which mean values and standard errors are reported.

Table [6](#_bookmark6) reports empirically estimated macro-centric entropy rates obtained from finite observa- tion windows of length *T ∈ {*15*,* 30*,* 90*}* days, together with the corresponding theoretical (ground- truth) entropy rates computed from the conditional micro-state Markov chains. For each macro- state, empirical entropy rates are estimated by restricting attention to windows fully contained within a macro-state sojourn and computing the plug-in entropy rate of the resulting micro-state transition process. Standard errors reflect variability across windows.

Table 6: Empirical macro-centric entropy rates (bits/day) for finite window lengths *T* , compared with theoretical values.

| macro-state | Theoretical | *T* = 15 days | *T* = 30 days | *T* = 90 days |
| --- | --- | --- | --- | --- |
| Acute (A) | 1.720 | 1*.*123 *±* 0*.*031 | 1*.*413 *±* 0*.*015 | 1*.*638 *±* 0*.*007 |
| Persistent (B) | 1.674 | 1*.*128 *±* 0*.*027 | 1*.*362 *±* 0*.*019 | 1*.*557 *±* 0*.*011 |
| Improving (C) | 1.128 | 0*.*925 *±* 0*.*032 | 0*.*954 *±* 0*.*014 | 1*.*040 *±* 0*.*007 |
| Stable well (D) | 0.632 | 0*.*749 *±* 0*.*037 | 0*.*691 *±* 0*.*014 | 0*.*613 *±* 0*.*005 |
| At-risk (E) | 1.582 | 1*.*015 *±* 0*.*018 | 1*.*251 *±* 0*.*010 | 1*.*465 *±* 0*.*006 |

Even for relatively short windows of *T* = 15 days, there is a clear and statistically robust separation between the *Stable well* and *Acute* macro-states, despite increased estimator bias and variance at short horizons. As the window length increases to *T* = 30 days, entropy-rate estimates stabilize and begin to separate intermediate macro-states, including *Improving* and *At-risk*. For longer windows (*T* = 90 days), empirical estimates closely approach the theoretical macro-centric entropy rates, and all five macro-states become clearly distinguishable.

This convergence behavior is consistent with the underlying time-scale separation in the model: typical macro-state sojourn lengths substantially exceed 30 days, enabling reliable estimation of third-order digital markers well before a macro transition occurs. These results confirm that macro- centric entropy rates are both theoretically well-defined and practically estimable from realistic observation horizons.

Numerical experiments indicate that even for relatively short windows of approximately *T* = 15 days, there is a clear separation between the stable well (D) and acute symptomatic (A) macro- states. For longer windows (*T ≥* 30 days), empirical entropy-rate estimates stabilize sufficiently

to distinguish all macro-states reliably. This confirms that third-order digital markers are not only theoretically well-founded, but also practically estimable from realistic observation horizons encountered in digital phenotyping studies.

**Real-time implementation and macro-state change detection:** In practical deployments, the patient’s macro-state is not directly observed and may evolve gradually rather than through abrupt transitions. Accordingly, entropy-rate estimation is performed continuously using a sliding window that advances day by day as new data become available. As the underlying macro-state begins to shift, the empirical entropy rate computed over the sliding window reflects a mixture of micro-dynamics from adjacent macro-states, resulting in smooth but systematic changes in the estimated value. Monitoring the temporal evolution of the window-based entropy rate therefore provides a natural mechanism for detecting macro-state transitions in real time. In particular, sustained deviations from baseline entropy levels or consistent trends toward the characteristic entropy range of another macro-state can be interpreted as early indicators of a regime shift. This continuous monitoring framework allows macro-state changes to be detected without explicit state labels, enabling adaptive, data-driven identification of clinically meaningful transitions as they unfold. In practice, standard change-point detection methods applied to the time series of sliding- window entropy-rate estimates can be used to automatically identify statistically significant shifts, providing a principled mechanism for detecting macro-state transitions in real time.

In a potential deployment, one practical issue will be that macro-states are not observed and patient-specific digital behavior exhibits stable idiosyncrasies. To solve this issue, one can use a hierarchical strategy: we initialize model structure and macro–micro templates from population-level priors, then rapidly personalize using each individual’s baseline data and update online as new observations arrive. Conceptually, the window-based entropy and micro-transition estimates become reliable once a window contains a sufficient number of within-macro micro-transitions. Empirically we found that windows on the order of a few weeks already separate stable-well from acute dysregulation, while longer windows improve differentiation among intermediate regimes. Prevention does not require observing multiple full episodes before deployment: change-point detection on the streaming entropy-rate estimate can flag statistically significant departures from the individual’s baseline, providing early warning even before the system can confidently assign a specific episode type. When clinically anchored events (e.g., clinician ratings, medication changes, hospitalization) occur, they can be used to refine macro-state templates over time, enabling progressively more specific prodrome detection (e.g., manic versus depressive), more personalized for a given patient.
